# Determining the Level to Affect of Physical Findings and Outcome Measures on Functional Status in Partial-Thickness Rotator Cuff Tears Using a Multiple Linear Regression Model

**DOI:** 10.3390/medicina62030574

**Published:** 2026-03-19

**Authors:** Ezgi Türkmen, İpek Yeldan, Nezih Ziroğlu, Süleyman Altun

**Affiliations:** 1 Clinical Research Coordination Unit, Health Institutes of Türkiye, Istanbul 34718, Turkey; ezgi.turkmen.49@hotmail.com; 2 Department of Physiotherapy and Rehabilitation, Institute of Graduate Studies, Istanbul University-Cerrahpaşa, Istanbul 34320, Turkey; 3 Division of Physiotherapy and Rehabilitation, Faculty of Health Sciences, Istanbul University-Cerrahpaşa, Istanbul 34320, Turkey; ipek.yeldan@iuc.edu.tr; 4 Department of Orthopedic Prosthetics and Orthotics, Vocational School of Health Services, Acibadem Mehmet Ali Aydinlar University, Istanbul 34638, Turkey; 5 Department of Orthopaedics and Traumatology, Acibadem University Atakent Hospital, Istanbul 34303, Turkey; 6 Clinic of Orthopaedics and Traumatology, Kanuni Sultan Suleyman Training and Research Hospital, University of Health Sciences, Istanbul 34303, Turkey; altun.suleyman@gmail.com

**Keywords:** functionality, multiple regression, correlation, pain, disability

## Abstract

*Background and Objectives*: It is crucial to determine physical findings and outcome measures that affect functional status of the patients, and the impact levels of these parameters on patients. Therefore, the aim of this study was to investigate the determinant and predictive effect of pain levels, shoulder range of motion (ROM) values, disability and health-related quality of life factors on functional status in individuals with partial-thickness rotator cuff tears (PRCT). *Materials and Methods*: Firstly, the functional status of 45 patients (mean age: 50.78 ± 5.28 years; 29 female) with PRCT, then activity and night pain levels with Numeric Pain Rating Scale, active flexion, abduction and external rotation of the shoulder ROM values with goniometer, disability level with Quick Disabilities of Arm, Shoulder & Hand Questionnaire, and health-related quality of life levels with Short Form-12 were evaluated and recorded. *Results*: It was detected that all determinants whose effect on functionality was evaluated with a multiple regression model explained 76% of the variance, and this effect level was statistically significant (R square = 0.760, adjusted R square = 0.707, F = 14.272, *p* < 0.001). Detailed evaluation showed that flexion and external rotation ROM values (respectively; β = 0.54, *p* < 0.001; β = 0.38, *p* = 0.001) and disability level (β = 0.44, *p* < 0.001) had statistically significant determinant effects on functional status. No statistically significant results which could be correlated with functional status were found for activity and night pain, abduction ROM value, and health-related quality of life domains (*p* > 0.05). *Conclusions*: Shoulder flexion and external rotation ROMs and disability level were found to have a predictive effect on the functional status in individuals with PRCT. It is noteworthy that more subjective and patient-reported findings and outcome measures such as pain and health-related quality of life had no predictive effect on functionality. By determining the level of these effects, results were reached that can shed light on the literature by guiding the development of reliable assessment algorithms.

## 1. Introduction

Partial-thickness rotator cuff tears (PRCT) represent a common clinical challenge, particularly in individuals engaged in repetitive overhead activities or those experiencing degenerative changes with age [1]. PRCT is a frequent cause of shoulder pain and dysfunction and accounts for a substantial proportion of rotator cuff pathology encountered in clinical practice. It has been reported that the incidence of the disease increases with age, with 6% of individuals under 60 years of age and 30% of individuals 60 years of age and older having the disease [2]. The effective management of PRCT depends heavily on the ability to correlate condition and functionality with physical findings and outcome measures. Although PRCTs are often considered less severe than full-thickness tears, longitudinal studies have reported that a considerable proportion may progress over time, leading to increased tear size, persistent symptoms, and functional deterioration if not appropriately managed [3,4]. Therefore, early identification of factors associated with functional limitation is of clinical importance. Examining the relationship between functional status with physical findings and outcome measures in PRCT is crucial to improve the effectiveness of treatment strategies.

The clinical and physical presentation of PRCT is heterogeneous and typically includes activity-related pain, night pain, limited shoulder range of motion (ROM), impaired upper-extremity function to disability and reduced quality of life [5]. Previous research has shown that shoulder pain intensity and night pain are strongly associated with functional disability and reduced participation in daily activities [6]. In addition, limitations in active ROM and deficits in muscle performance have been identified as key physical impairments contributing to activity restriction and decreased quality of life in patients with rotator cuff disorders [7]. Patient-reported outcome measures such as disability indices and health-related quality of life scales are therefore widely recommended to capture the multidimensional impact of shoulder pathology within the biopsychosocial framework [8].

Despite the growing body of evidence examining individual impairments, most previous studies have evaluated these factors separately or have focused on specific outcomes such as pain, or strength [9,10]. However, functional status in PRCT is likely influenced by the combined and interacting effects of multiple physical and patient-reported variables. Identifying the relative contribution of these factors using multivariate analytical approaches may provide more clinically meaningful information for assessment, prognosis, and treatment planning. To date, studies specifically investigating the simultaneous predictive effects of pain characteristics, shoulder ROM, disability, and health-related quality of life on functional status in individuals with PRCT remain limited.

Given that appropriate conservative management in the early stages may prevent disease progression, reduce the need for surgical intervention, and minimize the associated socioeconomic burden, a comprehensive understanding of determinants of functional limitation is essential. Also, understanding how patients’ functional status correlate with physical presentation (e.g., pain intensity, ROM) and outcome measures, such as disability and quality of life scales, is essential for developing reliable assessment algorithms and guiding evidence-based treatment decisions. Furthermore, the identification of physical findings or outcome measures associated with PRCT patients’ functional status can guide clinicians in stratifying patients according to severity and prognosis, thereby optimizing treatment choices. Recent studies emphasize the importance of a comprehensive, multimodal diagnostic approach that incorporates both subjective and objective measures [11,12]. Therefore, the aim of this study was to investigate the determinative and predictive effects of activity and night pain levels, shoulder ROM values, disability and health-related quality of life as independent variables on the dependent variable, functional status, in individuals with PRCT using a multiple linear regression model.

## 2. Materials and Methods

### 2.1. Trial Design

This study is a prospective, cross-sectional observational sub-study with preliminary evaluation data from a randomized controlled clinical trial. Study protocol was conducted in accordance with the “Declaration of Helsinki”, approved by the Clinical Research Ethics Committee of Istanbul University-Cerrahpaşa, and carried out in the Physiotherapy and Rehabilitation Department.

### 2.2. Participants

Participants who applied to the Department of Orthopedics and Traumatology between December 2021–July 2023 and were referred to us by an orthopedist with a diagnosis of PRCT tear through Magnetic Resonance Imaging and physical examination were included in the study.

The inclusion criteria for the study were as follows: people between the ages of 40–60 years, who were diagnosed with non-traumatic PRC tear by an orthopedist, had shoulder pain for more than 2 months, and volunteered to participate in the study.

Participants were excluded from the study if they had at least one of the following diagnoses or problems: full-thickness or massive RC tear, any other reported shoulder problem or previous surgery on treated shoulder, shoulder involvement due to any systemic or skin disease that would affect the upper-extremity evaluation, reported any diagnosed emotional or cognitive problem, and young and athletic participants with acute tear symptoms.

Informed consent was obtained from all participants, and information was given about the purpose of this study.

### 2.3. Sample Size Calculation

Sample calculation of the study was determined by the “G* Power 3.1 (Heinrich-Heine-Universität Düsseldorf, Düsseldorf, Germany)” program with linear multiple regression test [13]. Calculations were performed at 0.20 effect size, 0.05 significance level (α level 0.05), at a power level of 90%, with 8 predictor values. With the given parameters, the number of participants to be included in the study was determined as 45.

### 2.4. Outcome Measures

#### 2.4.1. Functional Status

*Shoulder Evaluation with Modified Constant-Murley Score*

Modified Constant-Murley (MCM) Score is a score that measures the functional status in shoulder injuries and combines objective and subjective features, providing an advantage to the evaluator by gathering all of the pain, loss of daily living activities, loss of movement and strength under a single roof. A maximum of “100” points can be obtained from the score, and high scores indicate good condition [14,15].

#### 2.4.2. Physical Findings

*Pain Intensity Assessment with Numeric Pain Rating Scale*

Shoulder pain levels felt during activity and at night were assessed with the 11-point Numeric Pain Rating Scale (NPRS). In this scale, a completely pain-free condition is defined as “0” and unbearable pain is defined as “10” [16].

*Goniometric Evaluation of Shoulder Joint Range of Motion*

ROM evaluations were performed by recording the active flexion, abduction, and external movements of the shoulder ROM at the pain limit. A digital goniometer (Baseline Evaluation Instrument^®^, Fabrication Enterprises, Inc., Elmsford, NY, USA) was used and the evaluations were made in accordance with the Kendall–McCreary protocol and criteria [17].

#### 2.4.3. Outcome Measures (Scales)

*Disability Assessment with Quick Disabilities of Arm, Shoulder & Hand Questionnaire*

The Quick version of the Disabilities of the Arm, Shoulder and Hand (Q-DASH) Questionnaire was used to evaluate the upper-extremity disability levels and functional status of the participants. The maximum score that can be obtained from the scale is determined as “100” and high scores are positively correlated with increased disability [18].

*Health-Related Quality of Life Assessment with Short Form-12*

Short Form-12 (SF-12) was used to evaluate the health-related quality of life. SF-12 consists of two subscales: the Physical Component Score (PCS-12) and the Mental Component Score (MCS-12). PCS-12 evaluates physical health status, whereas MCS-12 assesses mental well-being. The maximum score that can be obtained from the scale is determined as “100” and high scores are positively correlated with high health-related quality of life [19].

### 2.5. Study Organization

Within the scope of the study, firstly the dependent variable, “functional status,” was evaluated and recorded. Then, the independent variables; activity pain, night pain, flexion ROM, abduction ROM, external rotation ROM, Q-DASH score, PCS-12 and MCS-12 scores, whose effects on “functional status” were to be investigated, were evaluated and recorded. Physical findings included activity and night pain levels, active flexion, extension, and external rotation ROMs of the shoulder joint, while outcome measures were disability level and health-related quality of life. Evaluation of eligibility in terms of inclusion criteria (E.T.) and assessment of the outcome measures included in the study were performed by different researchers (İ.Y.).

Finally, multiple regression analysis of possible predictor physical findings and outcome measures that could be correlated with functional status was performed.

### 2.6. Statistical Analysis

Statistical analysis was performed using Statistical Package for Social Sciences version 27 for Mac OS. Descriptive statistics of the variables were expressed as mean ± standard deviation, and confidence intervals (CI). All analyzes were performed in the 95% CI, and *p* < 0.05 value was considered statistically significant.

Multiple linear regression test was used to test the influence of predictor variables on the dependent variable MCM score. In the interpretation of the results, collinearity variance inflation factors and tolerance values were taken into consideration and interpretation was made with *p* value and standardized coefficients beta value.

## 3. Results

A total of 45 participants (mean age: 50.78 ± 5.28 years; 29 female) who met the inclusion criteria were included in the analysis. None of the participants reported any negative events or side effects after assessments. Details of participants’ personal information, physical findings and mean values of outcome measures are presented in Table 1.

When the effect of the values that may have a determinant effect on the MCM Score was analyzed by multiple regression analysis, it was shown that all these predictors explained 76% of the variance and this effect level was found to be statistically significant (R square = 0.760, *p* < 0.001) (Table 2).

The results and effect levels of which of these values are effective on the MCM score are given in Table 3 in detail and below:

- A 1-unit standard deviation increase in flexion ROM value (9.48 degree) was found to produce a 54.5% standard deviation increase effect on the MCM score (the standard deviation of MCM score is 4.56; of which 54.5% corresponds to 2.48 points), holding other predictors constant (β = 0.545, *p* < 0.001). In other words, a 9.48-degree increase in flexion ROM was associated with a 2.48-point increase in the MCM score.
- A 1-unit standard deviation increase in external rotation ROM value (6.82 degree) was found to produce a 38% standard deviation increase effect on the MCM score (1.73 points) holding other predictors constant (β = 0.381, *p* = 0.001). A 6.82-degree increase in external rotation ROM was associated with a 1.73-point increase in the MCM score.
- A 1-unit standard deviation decrease in Q-DASH questionnaire value (7.43 points) was found to produce a 44% standard deviation increase effect on the MCM score (2 points) holding other predictors constant (β = 0.440, *p* < 0.001). A 7.43-point decrease in Q-DASH questionnaire score was associated with a 2-point increase in the MCM score.

No statistically significant predictive results could be correlated with functional status were found for NPRS activity and night levels, abduction ROM value, and health-related quality of life domains (*p* > 0.05).

## 4. Discussion

This study showed that shoulder flexion and external rotation ROM values and disability level had a total determinant effect of 76% on functional status in individuals with PRCT. These results are highly significant in terms of developing reliable assessment algorithms and guiding evidence-based treatment decisions in patients with PRCT. By identifying these physical findings and outcome measures associated with the functional status of PRCT patients, clinicians can be guided by the parameters that should be emphasized in the evaluation and thus optimize the treatment options. It is noteworthy that more subjective findings and patient-reported outcome measures, such as pain and health-related quality of life, which are frequently assessed in individuals with PRCT, did not have a predictive effect on functionality. The results of this study suggest that shoulder flexion and external rotation, along with disability level, are more decisive in the evaluation process than these subjective measures.

Although the clinical and physical presentation of PRCTs can vary, pain is the most common symptom. Shoulder pain may occur during active or passive arm elevation with a painful arc of motion, usually as a result of tendon degeneration and inadequate healing due to subacromial compression, peripheral and central sensitization, sensorimotor cortex changes or biochemical and patho-anatomical changes in RC tears [20,21]. Fukuda, H. et al. determined that 73% of persons diagnosed with partial-thickness rotator cuff tears had pain complaints above the moderate level according to a visual analog scale (>5) [22]. In this pathology group, where the level of pain can significantly affect the severity of the disease and the treatment algorithm, the effect of this pain level, which is especially aggravated by activity, on the general functional status of the patients is an important issue. As a result of the present study, as shown by the multiple regression results, it was observed that activity and night pain were not significant determining factors on the MCM score, which evaluates patients in detail at a functional status by gathering objective and subjective parameters under a single heading. While pain is an important physical finding for this patient group, this study demonstrates that pain is not a determinant finding that significantly impacts patient functional status. Although pain is a frequently reported symptom, its impact on functionality was found to be limited. The fact that pain is a self-reported, subjective assessment parameter may also have an impact on this result.

Decreased ROM, especially in overhead activities, may occur as a result of the combined symptoms such as pain intensity and pain avoidance, loss of strength and disability after PRCT [11]. Although this ROM reduction is expected to affect the level of functionality in daily life in PRCT patients, it is noteworthy that there is no data on the amount and effectiveness of this impact in the literature. The current study showed that shoulder flexion and external rotation ROM values are also significant predictive factors affecting the MCM score, which expresses functionality. Among the other findings and outcome measures evaluated, shoulder flexion ROM value was the factor with the most significant effect on the MCM score. When the shoulder abduction ROM value was examined, it was seen that although it had a certain level of effect on the MCM score, this effect did not reach the level of significance. The reason for this situation is that in the case of PRCT, the only muscle that provides abduction movement and force is not the supraspinatus, which is the most frequently torn muscle. More importantly, since the tearing of 1/3 or 2/3 of the supraspinatus tendon thickness has very little effect on force transmission, a severe weakness does not occur even though there is a decrease in ROM in abduction, and this does not significantly affect functionality [23,24].

As a result of rotator cuff tears, the concept of disability arises when individuals are unable to be away from their roles in daily life, their current jobs and social lives due to their symptoms or cannot be at the same level as their former healthy status [25]. Patients with PRCT experience disability as a result of combined physical findings such as pain, loss of shoulder ROM and strength, and this may result in a decrease in functional capacity in daily living activities [26]. It has been reported that one of the most frequently applied outcome measurements in RCT patients is the Q-DASH Questionnaire [27]. In the present study, we used the Q-DASH questionnaire to determine the disability levels of patients with PRCT and to determine the predictive effect of this level on the MCM score indicating functionality. Disability was found to be a significant influencing factor on the MCM score, as shown by the results of multiple regression analysis. This situation has shown us that the level of disability, which is also lacking in the literature, is a parameter that has a determining effect on functionality and should be prioritized in the evaluation and follow-up of PRCT patients.

The symptoms experienced due to rotator cuff tears affect the person’s perception of health over time [28,29]. It is stated that rotator cuff injuries lead to a decrease in health-related quality of life [26]. Piitulainen, K. et al. reported a correlation between reduced health-related quality of life and higher functional disability in individuals with RC injuries [30]. However, based on this correlation, it was not known whether health-related quality of life was an significant influencing factor on functionality and, if so, how much of a predictive effect it had. Based on multiple regression results, the current study showed that health-related quality of life is not a determining factor on the MCM score, which indicates the functional status of patients. Although the current literature indicates that the quality of life of patients with PRCT is affected, it is an important finding that this effect is not decisive on the functional status of patients. This situation has shown clinicians and academicians working with PRCT patients that subjective findings such as pain and quality of life assessed by the patient are not as decisive as objective outcome measures on the functionality that significantly affects the patient’s daily life.

From a clinical perspective, the present findings provide practical implications for patient assessment, stratification, and treatment planning in individuals with partial-thickness rotator cuff tears. The identification of shoulder flexion and external rotation ROM, together with disability level, as significant predictors of functional status suggests that clinicians should prioritize objective mobility assessment and functional disability evaluation during the initial examination. Patients presenting with marked limitations in flexion and external rotation ROM and higher disability scores may be considered at greater risk for functional impairment and may benefit from early, targeted rehabilitation programs focusing on mobility restoration, and functional training. Conversely, the lack of a predictive effect of pain intensity and health-related quality of life indicates that subjective symptom severity alone may not accurately reflect functional capacity in this population. These results support a stratified clinical approach in which treatment decisions are guided primarily by functional limitations and modifiable physical impairments rather than symptom perception alone. Such an approach may help clinicians identify patients who require more intensive conservative management, monitor treatment response using objective functional indicators, and optimize resource allocation by directing individualized, impairment-based rehabilitation strategies. Ultimately, integrating these key predictors into clinical evaluation may contribute to the development of more reliable assessment algorithms and facilitate evidence-based decision-making in the management of partial-thickness rotator cuff tears.

### Limitations

This study has some limitations that should be noted. The patient population referred to us with a diagnosis of PRCT is predominantly female. Although this is not a situation that can be intervened by us, it should not be forgotten that the results are determined on the basis of predominantly female gender.

Although the sample size is objectively determined through the software program, given the number of variables included, the sample size is relatively insufficient to meet the minimum assumptions required to run a regression model. Future studies with more participants would be more accurate in terms of the significance of the results.

Furthermore, the results of this study were derived from cross-sectional data; longitudinal studies are needed to understand the direction of the proposed correlations. Our study results will be the precursor of long-term studies in this respect.

## 5. Conclusions

As shown by the multiple linear regression results, this study concluded that the shoulder flexion and external rotation ROM values, and disability level had important predictive and influencing factor effect of 76% on the functional status in individuals with PRCT. Pain and health-related quality of life parameters, however, were found to have no significant predictive or influencing effect. It is noteworthy that more objective outcome measures such as shoulder ROM values and disability level have predictive and determinative effects on functional status when compared to subjective measures reported by the patient, such as pain and quality of life. By determining the predictive effects of flexion and external rotation ROM values and disability measure, which have been shown to have significant predictive effects on the functionality of PRCT patients, it will be possible to develop reliable assessment algorithms that prioritize these parameters, and this will shed light on the literature.

## Figures and Tables

**Table 1:** Participants’ demographic and baseline characteristics and complaint durations.

Assessments	Participants (n = 45) Mean ± SD
Age (year)	50.78 ± 5.28
BMI (kg/m^2^)	26.75 ± 4.10
Complaint Duration (months)	3.66 ± 2.64
Female/Male	29/16
Affected side (R/L)	29/16
Dominant side (R/L)	40/5
MCM Score	48.08 ± 4.56
NPRS Activity	7.24 ± 0.83
NPRS Night	6.76 ± 1.36
Shoulder ROM (°)	
*Flexion*	129.36 ± 9.48
*Abduction*	115.58 ± 11.11
*External Rotation*	62.33 ± 6.82
Quick DASH	46.34 ± 7.43
PCS-12	33.71 ± 4.77
MCS-12	45.45 ± 6.88

BMI: Body Mass Index; kg: kilogram; m: meter; R: Right; L: Left. MCM: Modified Constant Murley: Numeric Pain Rating Scale; DASH: Disabilities of the Arm, Shoulder and Hand; PCS-12: Physical Component Score-12; MCS-12: Mental Component Score-12; (°): Degree; SD: Standard Deviation. Independent-Samples T Test, *p* < 0.05. Chi-square test, *p* < 0.05.

**Table 2:** Multiple regression analysis results—model summary.

Model Summary				ANOVA ^a^	
***R Square***	***Adjusted R Square***	***F Change***	***Sig. F Change***	***F***	***Sig. ^b^***
0.760	0.707	14.272	<0.001	14.272	<0.001

^a^: Dependent Variable: Modified Constant Murley Score; ^b^: Predictors (NPRS Activity, NPRS Night, Flexion ROM, Abduction ROM, External Rotation ROM, Q-DASH, SF-12); *p* < 0.05.

**Table 3:** Multiple regression analysis results—coefficients.

Coefficients ^a^						
Independent Variables	Unstandardized B	Standardized Coefficients Beta	t	*p*	Collinearity Statistics	
					*Tolerance*	*VIF*
NPRS Activity	−0.136	−0.025	−0.258	0.79	0.719	1.391
NPRS Night	0.198	0.059	0.696	0.49	0.914	1.094
Flex ROM	0.263	0.545	4.130	**<0.001**	0.382	2.616
Abd ROM	0.020	0.049	0.365	0.71	0.371	2.692
ER ROM	0.255	0.381	3.579	**0.001**	0.588	1.701
Q-DASH	−0.270	−0.440	−4.845	**<0.001**	0.808	1.283
PCS-12	0.063	0.066	0.603	0.55	0.550	1.817
MCS-12	−0.093	−0.140	−1.241	0.22	0.524	1.909

^a^: Dependent Variable: Modified Constant Murley Score; VIF: Variance Inflation Factor; Numeric Pain Rating Scale; Flex: Flexion; Abd: Abduction; ER: External Rotation; Q-DASH: Quick Disabilities of the Arm, Shoulder and Hand; PCS-12: Physical Component Score-12; MCS-12: Mental Component Score-12; The bolded parts indicate statistically significant information, *p* < 0.05.

## Data Availability

This study is a prospective, cross-sectional observational sub-study that includes preliminary evaluation data from a randomized controlled clinical trial that is registered at ClinicalTrials.gov under registration number NCT05128474, and Registration Date: 22 October 2021.

## References

[1] Song A., Cannon D., Kim P., Ayers G.D., Gao C., Giri A., Jain N.B. (2022). Risk factors for degenerative, symptomatic rotator cuff tears: A case-control study. J. Shoulder Elb. Surg..

[2] Longo U.G., Berton A., Papapietro N., Maffulli N., Denaro V. (2012). Epidemiology, genetics and biological factors of rotator cuff tears. Med. Sport Sci..

[3] Keener J.D., Galatz L.M., Teefey S.A., Middleton W.D., Steger-May K., Stobbs-Cucchi G., Patton R., Yamaguchi K. (2015). A prospective evaluation of survivorship of asymptomatic degenerative rotator cuff tears. JBJS.

[4] Yamanaka K., Matsurnoto T. (1994). The Joint Side Tear of the Rotator Cuff: A Followup Study by Arthrography. Clin. Orthop. Relat. Res..

[5] Lafrance S., Charron M., Roy J.-S., Dyer J.-O., Frémont P., Dionne C.E., Macdermid J.C., Tousignant M., Rochette A., Doiron-Cadrin P. (2022). Diagnosing, managing, and supporting return to work of adults with rotator cuff disorders: A clinical practice guideline. J. Orthop. Sports Phys. Ther..

[6] Dunn W.R., Kuhn J.E., Sanders R., An Q., Baumgarten K.M., Bishop J.Y., Brophy R.H., Carey J.L., Holloway G.B., Jones G.L. (2014). Symptoms of pain do not correlate with rotator cuff tear severity: A cross-sectional study of 393 patients with a symptomatic atraumatic full-thickness rotator cuff tear. JBJS.

[7] Jeanfavre M., Husted S., Leff G. (2018). Exercise therapy in the non-operative treatment of full-thickness rotator cuff tears: A systematic review. Int. J. Sports Phys. Ther..

[8] Padua R., de Girolamo L., Grassi A., Cucchi D. (2021). Choosing patient-reported outcome measures for shoulder pathology. EFORT Open Rev..

[9] Kuhn J.E. (2009). Exercise in the treatment of rotator cuff impingement: A systematic review and a synthesized evidence-based rehabilitation protocol. J. Shoulder Elb. Surg..

[10] Chester R., Shepstone L., Daniell H., Sweeting D., Lewis J., Jerosch-Herold C. (2013). Predicting response to physiotherapy treatment for musculoskeletal shoulder pain: A systematic review. BMC Musculoskelet. Disord..

[11] Radhakrishnan R., Goh J., Tan A.H.C. (2023). Partial-thickness rotator cuff tears: A review of current literature on evaluation and management. Clin. Shoulder Elb..

[12] Bi A.S., Morgan A.M., O’Brien M., Waterman B.R., Strauss E.J., Golant A. (2024). Partial-Thickness Rotator Cuff Tears: Current Concepts. JBJS Rev..

[13] Faul F., Erdfelder E., Buchner A., Lang A.-G. (2009). Statistical power analyses using G* Power 3.1: Tests for correlation and regression analyses. Behav. Res. Methods.

[14] Çelik D. (2016). Turkish version of the modified Constant-Murley score and standardized test protocol: Reliability and validity. Acta Orthop. Traumatol. Turc..

[15] Fialka C., Oberleitner G., Stampfl P., Brannath W., Hexel M., Vécsei V. (2005). Modification of the Constant–Murley shoulder score—Introduction of the individual relative Constant score: Individual shoulder assessment. Injury.

[16] Michener L.A., Snyder A.R., Leggin B.G. (2011). Responsiveness of the numeric pain rating scale in patients with shoulder pain and the effect of surgical status. J. Sport Rehabil..

[17] Kendall F.P., McCreary E.K., Provance P.G., Rodgers M.M., Romani W.A. (2005). Muscles: Testing and Function with Posture and Pain.

[18] Chester R., Jerosch-Herold C., Lewis J., Shepstone L. (2017). The SPADI and QuickDASH are similarly responsive in patients undergoing physical therapy for shoulder pain. J. Orthop. Sports Phys. Ther..

[19] Ware J.E., Kosinski M., Keller S.D. (1996). A 12-Item Short-Form Health Survey: Construction of scales and preliminary tests of reliability and validity. Med. Care.

[20] Lewis J., McCreesh K., Roy J.-S., Ginn K. (2015). Rotator cuff tendinopathy: Navigating the diagnosis-management conundrum. J. Orthop. Sports Phys. Ther..

[21] Lam J.H., Bordoni B. (2023). Anatomy, shoulder and upper limb, arm abductor muscles. StatPearls [Internet].

[22] Fukuda H. (2000). Partial-thickness rotator cuff tears: A modern view on Codman’s classic. J. Shoulder Elb. Surg..

[23] Halder A., O’Driscoll S., Heers G., Mura N., Zobitz M., An K., Kreusch-Brinker R. (2002). Biomechanical comparison of effects of supraspinatus tendon detachments, tendon defects, and muscle retractions. JBJS.

[24] Shin S.J., Seo M.J. (2014). Partial thickness rotator cuff tears. Clin. Shoulder Elb..

[25] Razmjou H., Davis A.M., Jaglal S.B., Holtby R., Richards R.R. (2011). Disability and satisfaction after rotator cuff decompression or repair: A sex and gender analysis. BMC Musculoskelet. Disord..

[26] MacDermid J.C., Ramos J., Drosdowech D., Faber K., Patterson S. (2004). The impact of rotator cuff pathology on isometric and isokinetic strength, function, and quality of life. J. Shoulder Elb. Surg..

[27] Dabija D.I., Pennings J.S., Archer K.R., Ayers G.D., Higgins L.D., Kuhn J.E., Baumgarten K.M., Matzkin E., Jain N.B. (2019). Which is the best outcome measure for rotator cuff tears?. Clin. Orthop. Relat. Res..

[28] Gartsman G.M., Brinker M.R., Khan M., Karahan M. (1998). Self-assessment of general health status in patients with five common shoulder conditions. J. Shoulder Elb. Surg..

[29] Duckworth D.G., Smith K.L., Campbell B., Matsen F.A. (1999). Self-assessment questionnaires document substantial variability in the clinical expression of rotator cuff tears. J. Shoulder Elb. Surg..

[30] Piitulainen K., Ylinen J., Kautiainen H., Häkkinen A. (2012). The relationship between functional disability and health-related quality of life in patients with a rotator cuff tear. Disabil. Rehabil..

